# Utility of handheld non-mydriatic fundoscopy in a case of bilateral, reversible vision loss in an advanced HIV patient with cryptococcal meningitis in Sub-Saharan Africa

**DOI:** 10.1016/j.mmcr.2024.100680

**Published:** 2024-10-26

**Authors:** Kristoffer E. Leon, Timothy Mugabi, Tu Tran, Hawa Magembe, Caleb P. Skipper

**Affiliations:** a Department of Neurology, University of California, San Francisco, USA; b Infectious Diseases Institute, College of Health Sciences, Makerere University, Kampala, Uganda; c Department of Ophthalmology, University of Minnesota, MN, USA; d Department of Ophthalmology, College of Health Sciences, Makerere University, Kampala, Uganda; e Division of Infectious Diseases and International Medicine, Department of Medicine, University of Minnesota, Minneapolis, MN, USA

**Keywords:** Cryptococcus, Meningitis, Blindness, Fundoscopy, HIV

## Abstract

Cryptococcal meningitis is a fungal infection that is typically caused by *Cryptococcus neoformans* and most commonly seen in severely immunosuppressed patient. This disease causes severe neurologic disease due to elevated intracranial pressures. In this case report, we describe a patient with newly diagnosed HIV presenting to the hospital with cryptococcal meningitis complicated by sudden vision loss. We highlight the role of prompt diagnosis and treatment in the reversal of vision loss, with subsequent monitoring using a handheld, non-mydriatic fundus imaging device.

## 1 Introduction

Cryptococcal meningitis is a fungal infection typically caused by *Cryptococcus neoformans* and commonly seen in severely immunosuppressed patients and has significant morbidity and mortality [1,2]. In sub-Saharan Africa, cryptococcal meningitis is the most common cause of meningitis due to the significant burden of HIV disease [3]. Despite several recent randomized clinical [[4], [5], [6]] trials demonstrating important treatment advances for HIV-associated cryptococcal meningitis, many patients still suffer significant morbidity from the disease.

High intracranial pressure (ICP) is a common complication of cryptococcal meningitis and can lead to devastating sequelae. High ICP is thought to occur due to the sticky polysaccharide of the fungal capsule blocking cerebrospinal fluid (CSF) resorption through the arachnoid villi [7] Symptoms include headache, fever, neck pain, photophobia, confusion, nausea, and vomiting [8,9]. Vision loss is a devastating and fairly frequent complication of cryptococcal meningitis and is most commonly attributed to papilledema, infiltration of the optic nerve, and in rare cases, an optic nerve sheath compartment syndrome [7].

Cryptococcal meningitis is diagnosed through detection of the fungus from the CSF. Traditionally, this has been achieved with direct staining of the CSF with India Ink to visualize the encapsulated yeast cells [10] and performing fungal culture using fungal growth media. More recently, the development of the rapid, point-of-care diagnostics have made detection of cryptococcal meningitis far easier in resource limited settings. The CrAg LFA (IMMY; Norman, OK, USA) is a lateral flow assay for the detection of cryptococcal antigen from the blood or CSF. The test has excellent performance characteristics for the detection of *Cryptococcus* in advanced HIV disease, with published sensitivity of 99.3 % and specificity of 99.1 % [11]. Furthermore, the test is easy to use and gives results in 10 minutes. Incorporation of lateral flow assays and similar rapid diagnostics has dramatically improved the ability to diagnose cryptococcal meningitis in resource-limited settings.

Cryptococcal meningitis is initially treated with multi-agent antifungal induction therapy. In the African setting, traditionally amphotericin B deoxycholate (1mg/kg/day) and fluconazole (800–1200mg/day) were used in HIV-associated disease [12]. Recent trials have demonstrated good efficacy and reduced adverse events when liposomal amphotericin is combined with flucytosine and fluconazole (*5*). The World Health Organization (WHO) recently changed first-line treatment to a single high dose of liposomal amphotericin (10mg/kg once) combined with flucytosine (100mg/kg/day) and fluconazole (1200mg/day) for 14 days [13]. However, in addition to antifungal therapy, control of intracranial pressure has been associated with improved mortality [10]. Thus, serial therapeutic lumbar punctures are recommended in those with CSF opening pressures >250mm H_2_O, even in the absence of symptoms of ICP.

In this case report, we discuss a person living with HIV who presented to clinic in Kampala, Uganda with meningitis symptoms and acute vision loss. She was subsequently hospitalized and during management for her cryptococcal meningitis, she experienced intermittent vision changes. We highlight the use of a handheld fundus imaging device for monitoring disease progression and efficacy of our therapeutic interventions.

## 2 Case presentation

A 26-year-old woman newly diagnosed with HIV and started on antiretroviral therapy (ART) consisting of tenofovir, lamivudine, and dolutegravir three days prior presented with severe frontal headaches for one week, one episode of loss of consciousness (possible seizure), photophobia, dizziness, nausea, vomiting, neck and back pain, and confusion. Notably, the patient also presented with recent onset, bilateral, blurry vision loss that began 2 days prior to presentation and progressed to significant vision loss by time of admission.

On admission, her vital signs were most notable for mild hypertension with a blood pressure of 149/80 mm Hg, but otherwise was afebrile, with a normal heart rate of 85 beats per minute. Her exam initially had no focal neurologic deficits or meningismus and was most notable for dilated pupils that were minimally responsive to light. Her Glasgow Coma Scale (GCS) score was 14/15, with a point deducted for confusion and disorientation. The only lab available at this time was a CD4^+^ T cell count of 27 cells/μL, and a positive fingerstick (whole blood) cryptococcal antigen lateral flow assay (CrAg LFA) test.

**Fig. 1:**
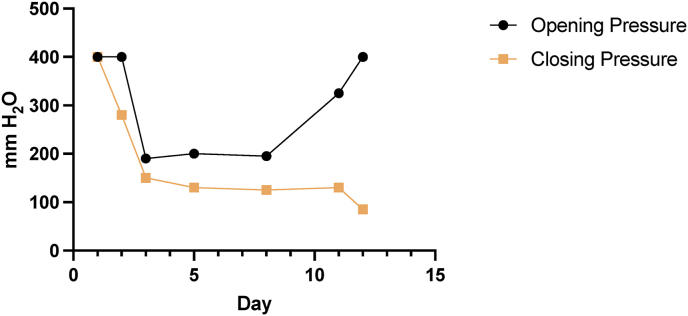
Opening and closing pressures in mmH20 for the diagnostic and therapeutic lumbar punctures by study day.

The patient underwent lumbar puncture (LP) due to concern for HIV-associated central nervous system infection. The initial LP found her opening pressure was >400 mm H_2_O (Fig. 1), with point-of-care testing demonstrating CSF glucose of 61 mg/dL, CSF lactate of 3.2 mmol/L, and a positive CSF CrAg LFA test. CSF analysis showed a turbid appearance, with proteins of 60 mg/dL, WBC of 60 cells/mm^3^ with a lymphocyte predominance, and visual identification of budding yeasts on Gram stain. Quantitative cryptococcal culture from this LP showed 84,500 CFU/mL. Approximately 32 mL of CSF were removed to normalize her intracranial pressure. Following the LP, the patient reported rapid improvement in her headache, and her vision improved from minimal perception (approximately hand motion) to identification of shapes and colors (approximately 20/800, though acuity was not formally tested). The pupils were dilated and minimally reactive without ability to discern an afferent pupillary defect in either eye. She was treated with a single dose of liposomal amphotericin B 10mg/kg, combined with flucytosine 100mg/kg/day and fluconazole 1200mg/day over 14 days for confirmed cryptococcal meningitis, consistent with World Health Organization (WHO) guidelines [13]. Her recently initiated ART was held, with plans to re-initiate 6 weeks after starting meningitis treatment (*14*).

**Fig. 2:**
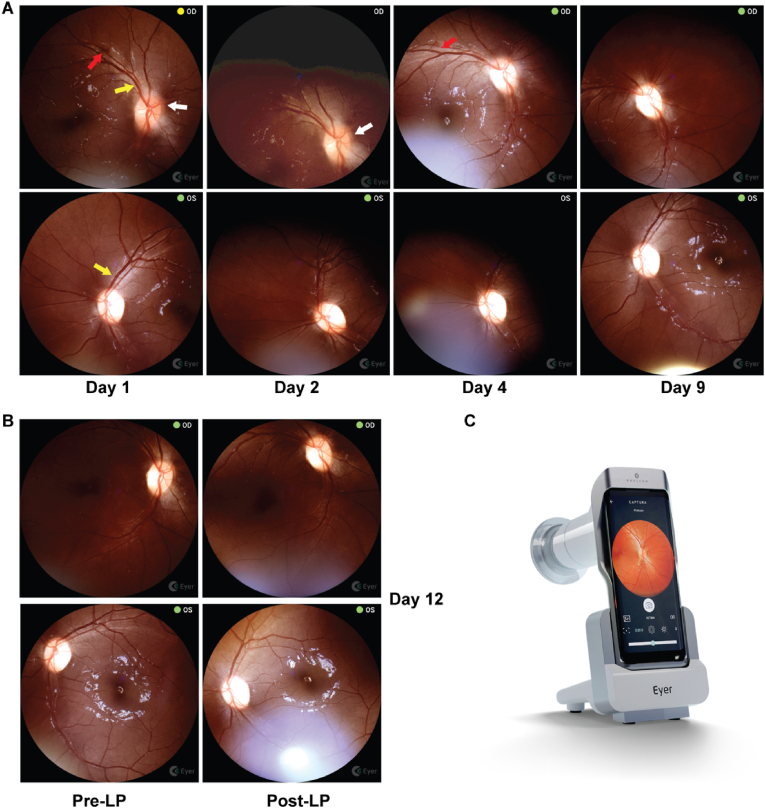
A) Serial retinal images on day 1 (presentation), day 2 (after first lumbar puncture), day 4 (after third lumbar puncture), and day 9 (after fifth lumbar puncture). Red arrows denote retinal hemorrhages, yellow arrows denote engorged retinal veins and white arrows highlight areas of blurring of the optic nerve head margin. The right eye had gliotic changes present and at most Frisen grade 1 papilledema with subtle blurring of the nasal margin with some involvement of the inferior and superior margins on presentation that resolved by day 4. The gliosis persisted and the nerve head had mild temporal pallor by day 9. The subtle segmental venous engorgement resolved rapidly by day 2 after the first lumbar puncture. The left eye notably had optic nerve pallor at presentation with one subtle segmental veinous engorgement of superotemporal vein that resolved by day 2. B) Mydriatic pre- and post-LP retinal imaging was performed on Day 12 after worsening of her visual symptoms. OD: right eye; OS: left eye. C) The Phelcom Eyer handheld non-mydriatic camera is pictured, utilizing a Samsung Galaxy S10 to capture images of the retinal fundus and upload them to an online image repository (*22*). (For interpretation of the references to color in this figure legend, the reader is referred to the Web version of this article.)

At this time, a non-mydriatic, handheld fundus imaging device (Phelcom Eyer fundus camera; Phelcom; São Carlos, Brazil) was utilized to establish a baseline fundus exam (Fig. 2A). The Eyer imaging device supports cloud-based storage and remote visualization of images, allowing for review by a trained reader. Fundus exam findings were also independently adjudicated by an in-person ophthalmologist who performed bedside fundoscopy. Her initial fundus exam demonstrated the right eye with chronic low-grade papilledema (gliotic changes, blurring of the optic disc margin, an engorged retinal vein and a single retinal hemorrhage). In the left eye, she had mild pallor of the optic disc. Otherwise there were no signs of vitreitis, no signs of CMV retinitis or cryptococcal choroiditis (based on expert review by trained ophthalmologists – both local and remote). The single hemorrhage was found to be resolving by day 4 (Fig. 2A, red arrow).

On day 2, she was found to have unilateral, left sided impairment of extraocular eye movement, left esotropia, ptosis, facial weakness, and difficulty swallowing. She also developed right sided hemiparesis of both the upper and lower extremities. Her right face and left extremities had full strength and sensation. Her headaches began to return, and her vision began to deteriorate, prompting the need for a second, therapeutic lumber puncture (LP). Again, she reported symptomatic and subjective vision improvement after the LP.

**Fig. 3:**
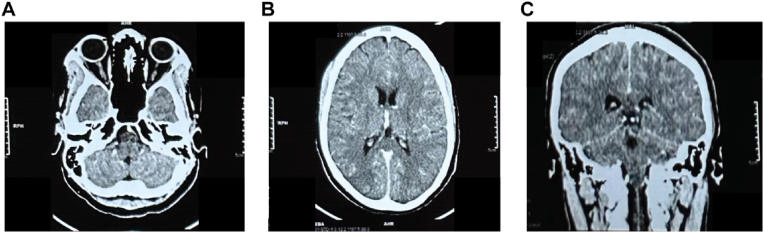
Contrasted head CT from day 3 showing A) no retinal hemorrhage; B) No large territory ischemia, intracerebral hemorrhage, mass effect, or hydrocephalus; C) herniation as an alternative explanation of acute vision loss.

Over the course of her illness, her headaches and vision continued a cycle of worsening, followed by improvement when therapeutic LPs were performed. After day 3, her opening and closing pressures normalized, but she still reported worsening visual symptoms that improved with a therapeutic LP. A contrasted head CT was obtained on day 5 and did not show any evidence of herniation, mass effect, hydrocephalus, hemorrhage, or ischemia (Fig. 3).

Her cranial nerve impairments and right sided hemiparesis improved as she continued antifungal therapy, and she was ambulatory by day 9. However, her course began to once again worsen, with decrease to light perception and recurrence of left eye abduction deficit, neck pain and hyperesthesia, and she was found to have a return of elevated ICPs (Fig. 1). She had a relative afferent pupillary defect in the right eye. She specifically reported that her vision on her left side was worse than on her right, with bilateral signs of nerve damage and loss of nerve fibers on fundoscopy (Fig. 2A). Therapeutic, large volume LPs were performed and symptoms rapidly improved. Mydriatic fundus imaging was performed before and after LP (Fig. 2B).

She was discharged on day 16 to complete the consolidation phase of 8 weeks of fluconazole 800 mg/kg/day and ART re-initiation at 6 weeks. Two weeks after discharge, patient reported significant improvement in symptoms over the phone with likely 20/800 vision in the right eye and hand motion in the left eye, although a mild headache was still persistent.

## 3 Discussion

Herein, we report an unusual case of abrupt visual loss, shortly after starting ART in a person with cryptococcal meningitis potentially partially accelerated and unmasked with ART initiation. Abrupt visual loss is unusual. In a 90 patient South African cohort, ophthalmological complications occurred in 46 % of patients, typically with a subacute to chronic loss of vision; interestingly, only 1 patient reported having sudden, profound vision loss like the patient reported here [7]. Cryptococcal-associated vision loss has been described as an initial AIDS presentation, similar to our patient who had just been diagnosed the day prior to symptom onset [14]. Other reports have highlighted that the presence of complete vision loss and similar neurological deficits are associated with poorer outcomes [15]. A previous report showed that 10 % of patients develop permanent vision loss [16]. Interestingly, vision loss in cryptococcosis has been described even with normal CSF opening pressures, which was seen over the latter portion of the hospitalization of our patient, and highlights the potentially multifactorial nature of this disease [17]. Multiple etiologies of vision loss may be present in cryptococcosis and include: central macular involving choroiditis, papilledema, optic nerve sheath compartment syndrome, and/or cortical visual impairment [7]. A multifactorial cause of out patient's visual loss is supported by her initial rapid improvement after lumbar puncture, followed by deterioration with normal opening pressures and then finally manifesting as disparate visual acuities after completion of induction treatment.

Furthermore, this case report highlights the utility of a handheld, non-mydriatic camera for fundal examinations performed by general medicine practitioners in a low-resource setting. In this study, we demonstrated that papilledema and subsequent improvement with normalization of CSF opening pressures is possible to monitor with a handheld device. The medicine practitioners used imaging to supplement their routine physical exam to guide management decisions on when to pursue therapeutic LP and motivated at least three of seven LPs. Importantly, using the mobile fundoscopy with tele-ophthalmology adjudication, we were able to rule out other causes of loss of vision loss, such as CMV retinitis, cryptococcal choroiditis or vitritis, or chorioretinitis. Since CMV retinitis is a treatable infection, time to diagnosis is an important factor in reducing morbidity and should be suspected in symptomatic patients with cryptococcosis.

Routine fundoscopy, especially in patients with either vision loss or neurological infections, should be performed to screen for early retinal or optic nerve pathology. In low resource settings, such as sub-Saharan Africa, where bedside ophthalmologic exams by a trained ophthalmologist can be difficult to obtain outside of a tertiary center, an easy to use, handheld non-mydriatic camera can help to fill in gaps of care and alleviate already overutilized specialty resources [18]. Furthermore, the cloud-based visualization of the images allows for deployment of these devices in locales where there are no ophthalmologists physically present, enabling tele-ophthalmological support. The Eyer device has been validated by comparison to standard fundus camera and used for diagnosis of diabetic retinopathy in rural Northern Brazil, highlighting the utility of these devices in areas without local ophthalmologic expertise [[19], [20], [21]].

Lastly, our case highlights the importance of CrAg screening in high burden populations. In general, persons with CD4 counts <100 cells/μL should undergo blood CrAg screening regardless of symptoms [13]. Our patient was seemingly already symptomatic with early cryptococcal meningitis when she was initiated on ART. Persons with any signs or symptoms of meningitis should receive a lumbar puncture and CSF testing for cryptococcal meningitis before starting antiretroviral therapy [13]. It is possible that the patient's complicated course with increased intracranial pressure and vision loss was accelerated/worsened in the setting of recently starting ART.

In summary, we present a case of HIV-associated cryptococcal meningitis complicated by raised intracranial pressure and vision loss. We demonstrate how mobile, non-mydriatic fundus cameras may be valuable in aiding the diagnoses of CNS complications in resource-limited settings, where trained ophthalmologists may not be available. Future studies testing the feasibility, diagnostic accuracy, and long-term outcomes of handheld fundus imaging utilizing a tele-ophthalmology approach are warranted.

## CRediT authorship contribution statement

**Kristoffer E. Leon:** Writing – original draft, Formal analysis, Data curation, Conceptualization. **Timothy Mugabi:** Writing – review & editing, Conceptualization. **Tu Tran:** Writing – review & editing, Formal analysis. **Hawa Magembe:** Writing – review & editing, Formal analysis. **Caleb P. Skipper:** Writing – original draft, Formal analysis, Data curation, Conceptualization.

## Declaration of competing interest

No potential conflicts of interest to report.
